# Modification of Human Umbilical Cord Blood Stem Cells Using Polyethylenimine Combined with Modified TAT Peptide to Enhance BMP-2 Production

**DOI:** 10.1155/2017/2971413

**Published:** 2017-08-17

**Authors:** Yufu Wang, Changcheng You, Rongzhi Wei, Jianing Zu, Chengchao Song, Jing Li, Jinglong Yan

**Affiliations:** ^1^Department of Orthopedics, Second Affiliated Hospital, Harbin Medical University, Harbin, China; ^2^Department of Pathology and Center of Electron Microscope, School of Basic Medicine, Harbin Medical University, Harbin, China

## Abstract

With the emerging role of umbilical cord blood-derived mesenchymal stem cells (hUCB-MSC) for bone regeneration and delivery of therapeutic proteins, there is an increasing need for effective gene delivery systems to modify such cells. mTAT, a TAT peptide sequence bearing histidine and cysteine residues, has been successfully used for intracellular gene delivery. Using a gWiz-GFP plasmid, we demonstrated that polyethylenimine combined with mTAT (mTAT/PEI) displayed good transfection efficacy in hUCB-MSC. hUCB-MSC transfected with mTAT/PEI were shown to express more BMP-2 protein and mRNA, indicating the feasibility of using the cells as a BMP-2 delivery system. Importantly, compared to PEI25, a “gold standard” nonviral transfection polymer, mTAT/PEI had limited toxicity to the cells. Furthermore, we demonstrated enhanced osteogenic activity in vitro for BMP-2 expressing hUCB-MSC. These results provide encouraging evidence for the potential use of mTAT/PEI to genetically modify hUCB-MSC as an approach to enhance tissue regeneration.

## 1. Introduction

Bone marrow is currently regarded as the main source of mesenchymal stem cells (MSC) as therapy for bone deficiencies [1]. However, the process of collecting bone marrow derived mesenchymal cells is invasive to donors and patients, and it can cause complications such as chronic pain, bleeding, and infection, thereby limiting the wide application of bone marrow-derived MSC in clinical settings [2]. Human umbilical cord blood contains many immature stem/progenitor cells with multilineage differentiation and extensive proliferation capacity and is considered an alternative source for stem cell-based therapies [1, 3]. Similar to bone marrow-derived MSC, human umbilical cord blood-derived mesenchymal stem cells (hUCB-MSC) also possess activity for immune modulation and tumor tropism [4, 5]. In addition, human hUCB-MSC can be obtained after full-term delivery of newborns, and the process of collecting hUCB-MSC is noninvasive and painless and does not harm the mother or infant. hUCB-MSC can be cryogenically stored, thawed, and expanded for therapeutic uses [6, 7], providing a convenient solution for the cells needed in therapy.

With the emerging role of hUCB-MSC as potential gene and cell therapy vehicles, there is an increasing need for effective gene delivery systems to modify such cells. The success of gene delivery is largely dependent on the development of a carrier that can efficiently transfer a target gene to hUCB-MSC with clinical safety and minimal toxicity [8]. Nonviral gene carriers are receiving increasing attention for the delivery of gene-based therapeutics because of their safety, flexibility in chemical design, and large capacity for vector delivery [9, 10]. The cationic polymer polyethylenimine (PEI) has been widely used for nonviral transfection and has an advantage over other polycations in that it combines strong DNA compaction with an intrinsic endosomolytic activity [11]. However, nonviral carriers are commonly less effective for gene transfer to stem cells when compared to viral delivery systems [12]. To develop a satisfactory gene carrier, various modifications have been made to PEI.

Cell-penetrating peptides (CPPs) have received great attention as efficient cellular delivery vectors [13]. Commonly amphipathic, rich in arginine, and positively charged, CPPs can deliver therapeutic and diagnostic molecules into cells in a nontoxic manner [14]. Several studies have shown the ability of CPPs to enhance polymer-mediated transfection in cell culture [15]. HIV-1 TAT peptide is the CPP most frequently used to deliver drugs, protein, and DNA into cells. Significant improvement in gene transfection efficiency has been demonstrated using a modified TAT peptide covalently fused with ten histidine and two cysteine residues when compared to unmodified TAT [16]. Moreover, it has been reported that the combination of mTAT with PEI could significantly improve transfection efficiency [17, 18].

In this paper, we report that mTAT/PEI/DNA complex displayed greater transfection efficiency over PEI25 and PEI for hUCB-MSC. Furthermore, the feasibility of BMP-2 expression and osteogenic differentiation was investigated in hUCB-MSC in vitro by using a custom-tailored expression vector (gWiz-BMP2) delivered by mTAT/PEI.

## 2. Materials and Methods

### 2.1. Materials

Branched PEIs of 2 kDa and 25 kDa were acquired from Sigma. mTAT, the HIV-1 TAT (RKKRRQRRRR) covalently fused with ten histidine and two cysteine residues (C-5H-Tat-5H-C), was synthesized and obtained from Changxi Biology Ltd. (Shanghai, China). The alkaline phosphatase (ALP) substrate p-nitrophenol phosphate (p-NPP), 8-hydroxyquinoline, o-cresolphthalein, 2-amino-2-methyl-propan-1-ol (AMP), dexamethasone, glycerophosphate, and ascorbic acid (AA) were also obtained from Sigma. The commercially available BMP-2 expression vector pCMV6-XL4-BMP2 was obtained from Origene (Rockville, MD). We obtained the blank (control) gWiz plasmid and its GFP expressing derivative gWiz-GFP from Aldevron Gene Therapy Systems, Inc. (San Diego, US). A CyQUANT cell proliferation kit was from Molecular Probes (Portland, OR). A BMP-2 ELISA kit was obtained from Wuhan Boster (Wuhan Boster, PR China). Phospho-Smad1/5/8 primary antibodies were purchased from Abcam (Cambridge, US).

### 2.2. Culture of hUCB-MSC

All procedures performed were in accordance with the ethical standards of the national regulations and with the 1964 Declaration of Helsinki and its later amendments or comparable ethical standards. Cord blood was collected with the mothers' informed consent in accordance with the guidelines of the Harbin Medical University Research Ethics Board. MSC were isolated and cultivated from human UCB as previously reported [19]. Light density mononuclear cells (MNC) were separated by density gradient centrifugation using Percoll (Amersham Biosciences, Uppsala, Sweden). MNC were cultured in Iscove's modified Dulbecco's medium (IMDM) supplemented with 10% fetal bovine serum (FBS) at 37°C in 5% CO_2_. After 24 h, nonadherent cells were removed and the complete medium was replaced. Cells were routinely passaged in Dulbecco's Modified Eagle Media (low glucose) with 10% FBS, 2 mmol/L L-glutamine and 1% penicillin-streptomycin. MSC from one CB sample at passages 4 to 8 were used in all the experiments. The identity of the MSC was confirmed by surface antigen expression of nonhematopoietic markers as described [15].

### 2.3. Transfection with Polymer Complexes

We constructed a BMP-2 expression vector by subcloning the BMP-2 fragment from pCMV6-XL4-BMP-2 into gWiz or gWiz-GFP. gWiz contains a modified promoter from the CMV immediate early gene promoter enhancer. This is done by restriction digest of plasmids with the NotI; digested gWiz was treated with Antarctic phosphatase to remove 5′-phosphate and minimize self-religation. Both the vector and insert DNA fragments were purified with the PCR Purification Kit from the enzymatic reactions before ligation with T4 DNA ligase. Then, the product was transformed to competent DH5*α* for amplification. Positive clones were further verified by restriction mapping with SalI, which generate asymmetric-size fragments that confirmed the directionality of the insert.

To prepare mTAT/DNA complexes, the peptide solution (1 mM) and the plasmid DNA were mixed in 5% glucose solution at pH 7 (final volume 10 mL/well), followed by quick vortexing for approximately 5 s. After the mixture was incubated at room temperature for 30 min, the cap of the sample tube was opened to expose the solution to air. The sample was shaken vigorously for 90 min. The cap was opened intermittently to allow air replenishment. With polymeric carriers (PEI2 or PEI25), the complexes were prepared in 150 mM NaCl at desired polymer : pDNA ratios and added to culture medium at indicated concentrations. Nontreated (NT) cells served as a control throughout the transfections. hUCB-MSC were plated in a 96-well cluster dish at a density of 2 × 10^4^ cells/mL, cultivated in DMEM containing 10% FBS. The cells were seeded 18 hours before transfection and culture medium was changed to fresh medium before complex addition. The indicated complexes (containing 2 *μ*g of plasmid DNA) were then added to the cells and incubated for 4–6 hours, after which the medium was changed to DMEM containing 10% FBS and antibiotics. GFP-positive cells were observed under a fluorescence microscope (Olympus, BX-60, Japan). The particle size and surface charge of complexes were measured by a Zetasizer Nano ZS instrument (Malvern Instruments, Worcestershire, UK) in triplicate at 25°C. The complexes containing 2 *μ*g of DNA at various weight ratios were diluted with NaCl solution to 1 mL. The size was presented as the average value of five runs.

### 2.4. Flow Cytometry for GFP Analysis

We trypsinized cells and fixed them with 3.7% formaldehyde in clear (i.e., without phenol red) HBSS. We quantified GFP expression with a Beckman Coulter QUANTA SC flow cytometer (Brea, CA) using the FL1 channel and the 488 nm blue laser. We calibrated instrument settings for each run to obtain a background level of GFP expression of 1% to 2% for the untreated control cells. The mean fluorescence per cell for the total population or GFP-positive cell population was also determined.

### 2.5. BMP-2 Protein Secretion by ELISA

The BMP-2 gene was delivered to hUCB-MSC. At 24 hours after transfection, the supernatant was collected for testing BMP-2 expression. Levels of BMP-2 production were evaluated by ELISA using corresponding anti-mouse antibodies and biotinylated secondary antibodies according to the manufacturer's instructions.

### 2.6. BMP-2 mRNA Expression by PCR

Total RNA was extracted from treated and untreated cultures using TRIzol reagent according to the manufacturer's protocol (Invitrogen). First-strand cDNA was synthesized using the PrimeScript RT-PCR reagent kit (Takara, China) according to the manufacturer's instructions. The specific primers used for BMP-2 were reverse primer, 5′-TCTCTGTTTCAGGCCGAACA-3′ and forward primer, 5′-TCTGACTGACCGCGTTACTC-3′. *β*-Actin was amplified as an internal control for normalization. PCR products were separated on 1.5% agarose gels and visualized by ethidium bromide staining, and the images were analyzed by the GEL DOC 2000 system (Bio-Rad, USA), where relative expression level (%) equaled gene band density divided by *β*-actin band density.

### 2.7. Western Blot Analysis

The BMP-2 downstream signaling pathway was investigated by Western blot analysis. Cells were lysed in immunoprecipitation buffer (50 mM Tris-HCl pH 7.5, 150 mM NaCl, 1% Triton X-100, 0.5% sodium deoxycholate) containing protease inhibitors. After centrifugation of the lysate, the supernatant was separated by SDS-PAGE and blotted onto a nitrocellulose membrane. The proteins were analyzed with anti-Smad1 and anti-phospho-Smad1/5/8 (Western blot) antibodies and visualized by SuperSignal West Femto Substrate system. Each blot presented was representative of the findings in at least three independent experiments. All signals were quantified by densitometry scanning.

### 2.8. MTT Assay for Cytotoxicity

Briefly, 100 *μ*L of MTT solution (5 mg/mL) was added to each well containing cells with 0.5 mL medium. After 2 hours, the medium was removed, and 500 *μ*L DMSO was added to the wells to dissolve the formed MTT formazan crystals. The absorbance was then measured at 570 nm and used as a relative measure of total cell activity. The MTT absorbance of transfected cells was compared to that of nontreated (NT) controls (100% viability), and the viability of transfected cells was expressed as percentage of nontreated cells. MTT assays were performed at 24, 48, and 72 hours after transfection.

### 2.9. Analysis of Osteogenic Differentiation

The DNA content, ALP activity and calcification in cells were determined as a measure of osteogenic activity. The cells were grown in 24-well plates for this purpose and transfected with desired complexes. After complex removal, the cells were cultured in osteogenic medium (DMEM supplemented with 10% FBS, 10 nM dexamethasone, 10 mM *β*-glycerophosphate, and 50 mg/L ascorbic acid). At the indicated times, the cells were washed with HBSS (×2) and lysed with ALP buffer (0.5 M 2-amino-2-methylpropan-1-ol and 0.1% Triton X; pH: 10.5) at 4°C. After 2 hours, 150 *μ*L of lysed solution was transferred into 48-well plates, and 150 *μ*L of 2 mg/mL ALP substrate (p-NPP) was added to the cell lysate. The kinetics of absorbance change (at 405 nm) were immediately determined at 405 nm for up to 15 minutes. The remaining cell lysis solution was frozen at −20°C and thawed at a suitable time for DNA analysis. The CyQUANT DNA kit was used for DNA analysis according to the manufacturer's instructions (*λ*_abs_ = 480 nm, *λ*_em_ = 527 nm). A DNA standard provided by the CyQUANT kit was used to estimate the DNA concentration, which was used to normalize the ALP activity obtained above.

Calcium deposits were stained by Alizarin Red S staining and quantified at approximately 21 days. Briefly, cells were washed with PBS and fixed with 10% formalin for 30 min. Alizarin Red S staining working solution with pH value approximately 4.1–4.3 was used to stain the cells. Thoroughly washed with distilled water, calcium deposits with red color could be observed under a microscope. Quantification of calcium deposition was made by extracting Alizarin Red S staining with 10% cetylpyridinium chloride (CPC) and measuring the absorbance at 570 nm.

### 2.10. Statistical Analysis

The results were expressed as the mean ± standard deviation (SD) of triplicate experiments. Similar results were obtained at least two times. Data analysis was performed by one-way analysis of variance (ANOVA) followed by Tukey post hoc testing with SPSS software package (SPSS, Chicago, IL, USA). Statistical significance was determined by *p* values less than 0.05.

## 3. Results

### 3.1. Efficiency of Gene Delivery to hUCB-MSC Using mTAT/PEI/DNA Complex

**Figure 1:**
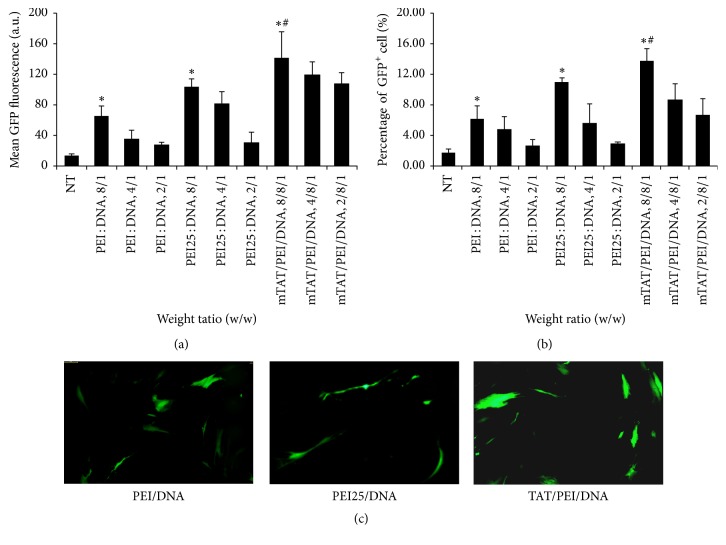
Transfection efficiency of mTAT/PEI carriers in hUCB-MSC. The cells were transfected with gWiz-GFP complexes of PEI, PEI25, and mTAT/PEI. GFP expression was analyzed by flow cytometry 24 hours after transfection. The weight ratio (polymer/gWizDNA or mTAT/polymer/gWizDNA) used for transfection is indicated in the horizontal axis. (a) Mean GFP fluorescence induced in the cell population. (b) GFP-positive cell population in percentage. (c) Fluorescence images using gWiz-GFP complexes of PEI (polymer/gWizDNA weight ratio at 8/1), PEI25 (polymer/gWizDNA weight ratio at 8/1), or mTAT/PEI (mTAT/polymer/gWizDNA weight ratio at 8/8/1). ^*∗*^*p* < 0.05 versus NT. ^#^*p* < 0.05 versus PEI or PEI25.

After 24 h of transfection, the transfection efficiency was evaluated using flow cytometry. Both mTAT/PEI, PEI, and PEI25 resulted in significantly increased GFP expression in hUCB-MSC. mTAT/PEI showed significantly higher mean GFP fluorescence than PEI25 or PEI (Figure 1(a)). mTAT/PEI also triggered a higher percentage of GFP-positive cells compared to PEI25 or PEI (Figure 1(b)). Optimal transfection was dependent on the polymer/DNA weight ratio for PEI and PEI25, both being achieved at polymer/pDNA weight ratio of 8 : 1. This ratio was used for analyzing the optimal mTAT : PEI : DNA ratio. Optimal transfection conditions were evaluated by varying the amount of mTAT for different mTAT/PEI ratios. Optimal transfection efficiency was achieved with an mTAT : PEI : DNA weight ratio of 8 : 8 : 1. These conditions were selected for further transfection studies. Fluorescence images showed more transfected GFP-positive cells when using the mTAT : PEI : DNA complex weight ratio of 8 : 8 : 1.

### 3.2. Effect of mTAT/PEI/DNA BMP-2 on Particle Charge and Size

**Figure 2:**
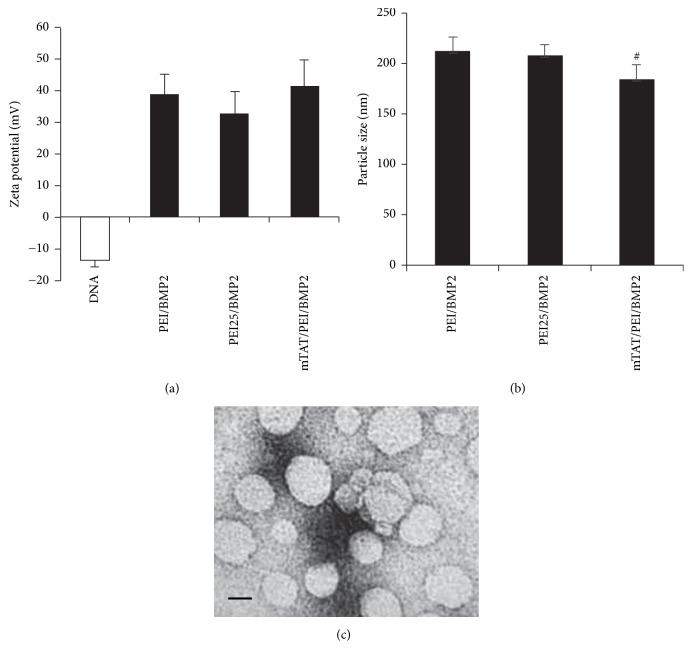
Characterization of PEI (polymer/gWizDNA weight ratio at 8/1), PEI25 (polymer/gWizDNA weight ratio at 8/1), and mTAT/PEI (mTAT/polymer/gWizDNA weight ratio at 8/8/1) complexes of gWiz-BMP2. (a) Zeta potential of the three complexes' formulations (Figure 2(a)). (b) Nanoparticle size of the two complexes' formulations (Figure 2(b)). (c) Electron microscopic image of mTAT/PEI/DNA complexes (mTAT/polymer/gWizDNA weight ratio at 8/8/1). Scale bar, 100 nm. ^#^*p* < 0.05 versus PEI or PEI25.

The zeta potential and particle size of the complexed particles were determined. The zeta potential of the DNA alone was highly negative. The zeta potential rose to over 30 mV when DNA was combined with mTAT/PEI, PEI25, and PEI. The most positive value of the zeta potential was for mTAT/PEI/DNA (Figure 2(a)). The *z*-average diameter was significantly smaller for mTAT/PEI than PEI25/DNA and PEI/DNA (Figure 2(b)). The formed complexes of mTAT/PEI/DNA appeared as nanoparticles with a spherical shape and compacted structure, as shown in TEM images (Figure 2(c)). The TEM images of PEI25/DNA and PEI/DNA are shown in Supplementary Material available online at https://doi.org/10.1155/2017/2971413.

### 3.3. Cell Viability of hUCB-MSC Transfected with mTAT/PEI/BMP2 Complex

**Figure 3:**
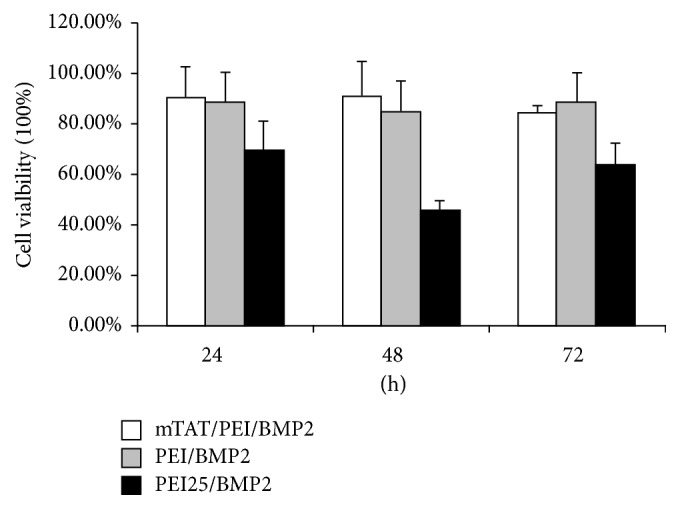
Viability of hUCB-MSC treated with PEI, PEI25, or mTAT/PEI complexes of gWiz-BMP2. The MTT assays were performed 24, 48, and 72 hours after transfection, and the cell viability obtained was expressed as a percentage of nontreated cells (taken as 100% viability).

Cell viability was evaluated by the MTT assay at 24, 48, and 72 h after BMP-2 gene transfection. The cell viability was expressed as a percentage of the untreated cell controls (Figure 3). Treatment of the cells with PEI25 complexes led to a dramatically decreased growth trend at 24 and 48 h, after which the cell viability was slightly increased at 72 h. However, mTAT/PEI was not obviously toxic to hUCB-MSC during the study period.

### 3.4. BMP-2 Secretion by hUCB-MSC Transfected with mTAT/PEI BMP-2 Complex

**Figure 4:**
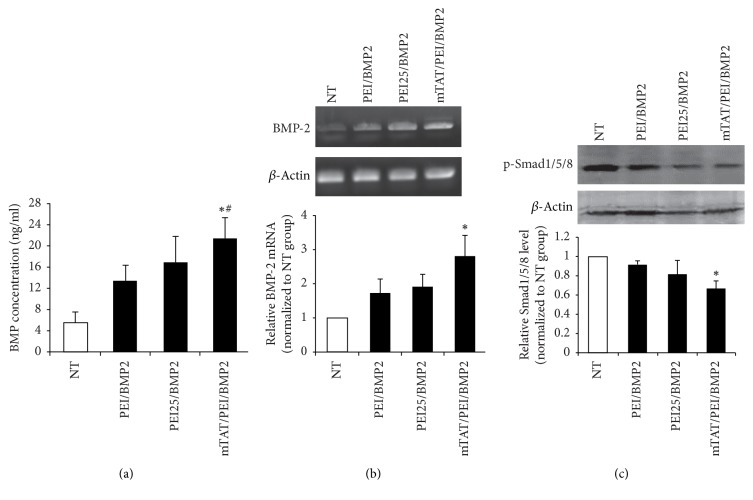
Protein and mRNA expression of BMP-2 from hUCB-MSC modified with gWiz-BMP-2 complexes of mTAT/PEI, PEI25, and PEI. The culture supernatant and RNA samples were each collected at 2 days after transfection. (a) BMP-2 concentration in the supernatant was analyzed by ELISA. (b) BMP-2 mRNA expression was determined by RT-PCR. (c) Activation of its downstream Smad1/5/8 signaling was studied through Western blot analysis. ^*∗*^*p* < 0.05 versus NT. ^#^*p* < 0.05 versus PEI or PEI25.

Strong production of BMP-2 was detected following plasmid transfer to hUCB-MSC using PEI25 and mTAT/PEI, where transfection with mTAT/PEI/BMP2 complex resulted in the highest increase of BMP-2 secretion (Figure 4(a)). RT-PCR analysis revealed elevated mRNA expression in transfected cells using the three complexes. More significant BMP-2 mRNA expression was detected in the mTAT/PEI delivery group when compared to the nontreated and PEI25 delivery groups (Figure 4(b)). In addition, the results of a Western blot assay showed that activated Smad1/5/8 in the phosphorylated form was enhanced in mTAT/PEI/BMP2 transfected cells compared with the other two groups (Figure 4(c)).

### 3.5. ALP Activity of hUCB-MSC Transfected with mTAT/PEI/BMP2 Complex

**Figure 5:**
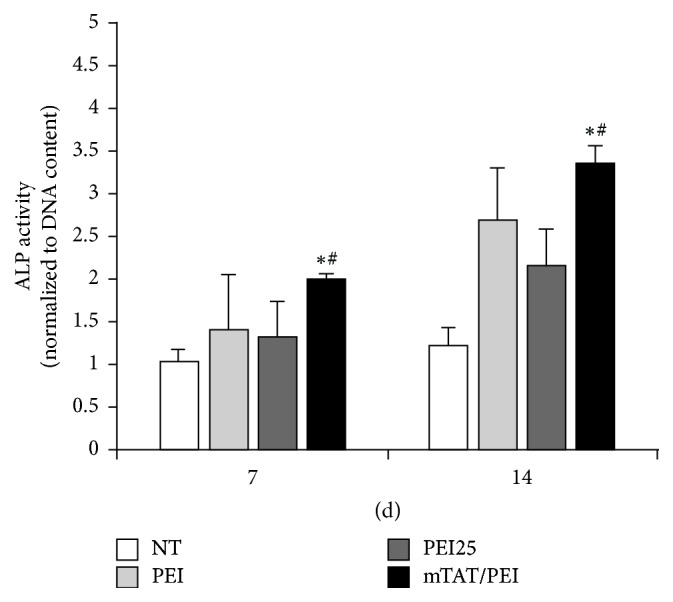
Specific ALP activity of CB-MSC treated with PEI, PEI25, or mTat/PEI complexes of gWiz-BMP2. Transfection with complexes was carried out for 24 hours, after which the medium was changed to OM. The DNA content and ALP activity were determined at 7 and 14 days after transfection. ^*∗*^*p* < 0.05 versus NT. ^#^*p* < 0.05 versus PEI or PEI25.

Since ALP is considered an early marker of osteoblast differentiation, the ALP activity of hUCB-MSC was evaluated 7 days and 14 days after transfection. The cells transfected with mTAT/PEI/BMP-2 complexes expressed significantly higher levels of ALP activity than the PEI25 and nontreated groups (Figure 5).

### 3.6. Calcification of hUCB-MSC Transfected with mTAT/PEI/BMP2 Complex

**Figure 6:**
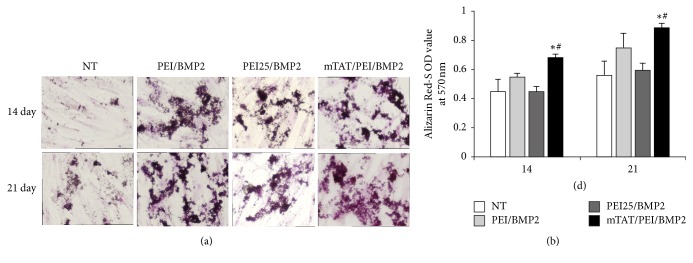
Analysis of calcification in hUCB-MSC cultures treated with PEI, PEI25, or mTAT/PEI complexes of gWiz-BMP2. Transfection with complexes was carried out for 24 hours, after which the medium was changed to OM. The extent of calcification was quantified spectroscopically at 14 and 21 days after transfection. (a) Representative Alizarin Red-stained cultures. (b) Quantitative analysis of calcium deposition. ^*∗*^*p* < 0.05 versus NT. ^#^*p* < 0.05 versus PEI or PEI25.

A significant amount of calcification was seen with hUCB-MSC transfected with mTAT/PEI/BMP2 complexes at 14 days after transduction. The untreated cells exhibited minimal calcium deposition at that time. At 21 days after transfection, BMP-2 transfected hUCB-MSC displayed a marked elevation of calcium deposition. Alizarin Red S staining on day 21 also revealed a significant increase of calcium nodules in hUCB-MSC transfected with mTAT/PEI, where nontreated cells displayed less calcification (Figure 6(a)). The quantification of osteogenic differentiation was also evaluated by extracting the staining using CPC solution to calculate the calcium deposition (Figure 6(b)).

## 4. Discussion

The unique differentiating features of mesenchymal stem cells can help to improve the current treatment for various diseases and provide functional tissues to repair or even replace diseased tissues. Mesenchymal stem cells can be obtained from various sources, for example, bone marrow, skin, and fat tissue. It has been demonstrated that the use of adult tissue-derived mesenchymal stem cells might not always be acceptable because of a significant drop in pluripotent cell numbers and proliferative/differentiation capacity with age [20]. Therefore, the search for alternative sources of BMSC is of significant value. Umbilical cord blood has turned out to be a viable alternative source of MSC for clinical gene and cell-based therapies. hUCB-MSC have been demonstrated to be capable of differentiating into osteoblasts, adipocytes, neural cells, and skeletal myoblasts [2, 21, 22]. Studies have also shown that hUCB-MSC exhibit a greater proliferative capacity when compared with bone marrow derived MSC and do not experience contact-inhibited cell growth, unlike BMSC, which fail to continue to proliferate after reaching 100% surface confluence [21].

Our data showed that transfected hUCB-MSC undergo rapid osteogenesis based on the increase in ALP activity and calcium deposition within 14 days. This osteogenesis of BMP-2 transfected hUCB-MSC is faster than the reported osteogenesis rate of bone marrow-derived MSC, which usually take >4 weeks for robust calcification. On the one hand, the rapid osteogenesis of transfected hUCB-MSC may be the result of the higher transfection efficacy and the higher BMP-2 secretion. On the other hand, this may be attributed to the fact that the hUCB-MSC have a higher percentage of osteoprogenitor stem cells [23, 24]. Other studies also reported that hUCB-MSC had significantly stronger osteogenic potential [25].

In this study, we found transfection efficiency of PEI/DNA complexes at N/P: 8 : 1, suggesting that PEI is an effective gene delivery system for hUCB-MSC. Moreover, we observed that the transfection efficiency of mTAT/PEI/DNA complexes could be further enhanced by the appropriate choice of weight ratio. These data indicated that mTAT combined with PEI enhanced transfection efficiency in vitro. For effective transfection, the first step is to have an electrostatic interaction between the carrier/DNA complex and cell membrane. TAT might lead to particles more suitable for membrane penetration [26]. It has also been reported that polymerization of TAT by disulfide bonds or tandem peptide bonds may stabilize particles and/or facilitate interaction with the cell membrane. CPPs can overcome the poor permeability of biological molecules through the plasma membrane of cells via adsorption to glycosaminoglycans of the cell membrane and processing through the endocytic mechanism. CPPs with small cargo may enter cells quickly via direct translocation in addition to the endocytic way [14].

Moreover, the positively charged structure of the TAT/PEI/DNA complex and its size played important roles in determining successful gene delivery [27, 28]. We investigated the transfection ability of hUCB-MSC using synthesized mTAT/PEI/DNA, which gave higher transfection than PEI25 and PEI, and more importantly, with little toxicity. The higher transfection performance of mTAT/PEI/DNA may be attributed to the hydrophobic character and cell-penetrating property of the nanoparticles. Hydrophobic character may have several effects on several key steps of gene transfer such as DNA condensation, cellular interaction and uptake, endosomal escape, and unpacking of polyplexes [29–31]. A positively charged structure is desirable since it can preferentially adhere to the negatively charged cell surface receptors, leading to endocytosis. Uptake of large molecules attached to these peptides tends to be mediated by macropinocytosis in an energy-dependent manner with slower rates for larger compounds [14, 15]. Previous studies suggest that the particle size of the mTAT/PEI/DNA complex (approximately 180 nm) may be the optimal size for internalization, and mTAT/PEI/DNA might be internalized by caveolae-mediated endocytosis [17]. It is not surprising to see that osteogenesis (based on ALP activity and calcification) was enhanced at the higher mTAT/PEI/DNA weight ratio, where increased transfection with the BMP-2 expression vector was observed. This was directly demonstrated by assessing the level of BMP-2 protein secreted into the medium. Although a level equivalent to PEI25 was obtained, the main reason for this was the protein released from lysed cells in cultures that upregulated the BMP-2 concentration in supernatant.

## 5. Conclusions

Collectively, we have shown that mTAT/PEI/DNA is an efficient nonviral carrier for hUCB-MSC modification, affording nontoxic and superior nonviral gene transfection compared to the 25 kDa PEI control. The successful BMP-2 expression in vitro upon modification of hUCB-MSC with mTAT/PEI/BMP2 offers promise for CPP-polymer-mediated gene delivery and providing potential applications in stem cell-based gene therapy.

## Supplementary Material

The representative TEM images of PEI25/DNA and PEI/DNA.
