# Supplementary material for: Modification of Human Umbilical Cord Blood Stem Cells Using Polyethylenimine Combined with Modified TAT Peptide to Enhance BMP-2 Production
Source: Biomed Res Int. 2017 Aug 17;2017:2971413. doi: 10.1155/2017/2971413 (PMC5603109; doi:10.1155/2017/2971413)
Supplement: Supplementary file 1 — The representative TEM images of PEI25/DNA and PEI/DNA. [file 2971413.f1.docx]

**Supplementary file：**

**Electron microscopic image of PEI25/DNA and PEI/DNA complexes**

Scale bar 100nm


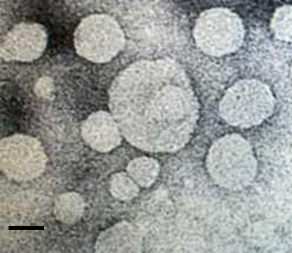


**PEI25/DNA**


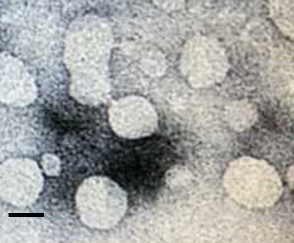


**PEI/DNA**
